# Efficacy of Intensive Control of Glucose in Stroke Prevention: A Meta-Analysis of Data from 59197 Participants in 9 Randomized Controlled Trials

**DOI:** 10.1371/journal.pone.0054465

**Published:** 2013-01-23

**Authors:** Chi Zhang, Yu-Hao Zhou, Chun-Li Xu, Feng-Ling Chi, Hai-Ning Ju

**Affiliations:** 1 Department of Neurosurgery, Shanghai Seventh People’s Hospital, Shanghai, People’s Republic of China; 2 Department of Rehabilitation Institute, Shanghai Seventh People’s Hospital, Shanghai, People’s Republic of China; 3 Department of Neurology, Shanghai Seventh People’s Hospital, Shanghai, People’s Republic of China; 4 Department of Cardiology, Shanghai Seventh People’s Hospital, Shanghai, People’s Republic of China; Universidad Peruana Cayetano Heredia, Peru

## Abstract

**Background:**

The efficacy of treatments that lower glucose in reducing the risk of incident stroke remains unclear. We therefore did a systematic review and meta-analysis to evaluate the efficacy of intensive control of glucose in the prevention of stroke.

**Methodology/Principal Findings:**

We systematically searched Medline, EmBase, and the Cochrane Library for trials published between 1950 and June, 2012. We included randomized controlled trials that reported on the effects of intensive control of glucose on incident stroke compared with standard care. Summary estimates of relative risk (RR) reductions were calculated with a random effects model, and the analysis was further stratified by factors that could affect the treatment effects. Of 649 identified studies, we included nine relevant trials, which provided data for 59197 patients and 2037 events of stroke. Overall, intensive control of glucose as compared to standard care had no effect on incident stroke (RR, 0.96; 95%CI 0.88–1.06; P = 0.445). In the stratified analyses, a beneficial effect was seen in those trials when body mass index (BMI) more than 30 (RR, 0.86; 95%CI: 0.75–0.99; P = 0.041). No other significant differences were detected between the effect of intensive control of glucose and standard care when based on other subset factors.

**Conclusions/Significance:**

Our study indicated intensive control of glucose can effectively reduce the risk of incident stroke when patients with BMI more than 30.

## Introduction

Cardiovascular disease is the leading cause of premature morbidity and mortality in the developed world, and it has emerged as one of the leading causes in developing countries such as China [1], [2]. Previous meta-analysis [3] have already provided a clear evidence of the role that glucose have in the causation of vascular disease, which indicated that raised concentrations of glucose in blood have been suggested to be a modifiable, independent risk factor for coronary heart disease and myocardial infarction. However, the efficacy of treatments that lower glucose concentration in reducing the risk of incident stroke has not been confirmed by randomized controlled trials and meta-analysis.

There are several possible reasons for the inconsistent findings between the recent randomized controlled trials and earlier observational studies. Firstly, individual trials might have been underpowered to show clinical benefit, especially if event rates were lower than were expected because of improved control of risk factors; Secondly, the relationship between glucose levels and incident stroke was described initially by observational studies, which may overestimate the effect of this relationship. Finally, duration of treatment was shorter than was needed to show a clinical benefit, or differences in glucose control between patients group were to small to show any benefit.

For a better understanding of the efficacy of glucose control on incident stroke, data from recent trials need to be re-evaluated and combined with data in former literature. Therefore, we carried out a systematic review and meta-analysis of pooled data from randomized controlled trials focusing on incident stroke as the disease endpoint in relation to lower glucose.

## Methods

### Data Sources, Search Strategy, and Selection Criteria

Randomized controlled trials of patients either to an intersive control of glucose versus a standard regimen (placebo, standard care, or glucose control of reduced intensity) in English-language were eligible for inclusion in our meta-analysis. Relevant trials were identified with the following procedure:

- Electronic searches: we searched Medline, EmBase, and the Cochrane Library for trials published between 1950 and June, 2012, with terms related to glucose and stroke (“stroke”, “glucose”, “diabetes mellitus”, “human”, “English”, and “randomized controlled trials”). All reference lists from reports on non-randomized controlled trials were searched manually for additional eligible studies.
- Other sources: we contacted authors to obtain any possible additional published or unpublished data and we searched http://www.ClinicalTrials.gov for information on registered randomized controlled to identify trials that were registered as completed but not yet published.

The literature search, data extraction, and quality assessment were undertaken independently by two authors (CZ and CLX) with a standardized approach, and any disagreement between these two authors was settled by a third author (YHZ) until a consensus was reached. We restricted our study to randomized controlled trials, which are less likely to be subject to confounding biases than are observational studies. Study were eligible for inclusion if: (1). The study was a randomized controlled trials; (2). The number of events for stroke that occurred during the study more than ten incident cases; (3). The trials assessing the effects of intensive control of glucose compared with standard care; (4). The duration of follow-up was at least 12 months. This review was conducted and reported according to the PRISMA (Preferred Reporting Items for Systematic Reviews and Meta-Analysis) Statement issued in 2009 (Table S1) [4].

### Data Collection and Quality Assessment

Two reviewer (FLC and HNJ) gathered information in duplicate using a standardized format from all relevant studies, and the third author (YHZ) adjudicated any discrepancies. Recorded data variables were as follows: first author or study group, publication year, number of patients, percentage male, mean age, body mass index (BMI), total cholesterol, glycosylated hemoglobin, patients current disease, intervention regimes, type of control, duration of follow-up, and number of incident stroke for each treatment group. We also measured the quality of the trials included in this study with the Jadad score [5] based on randomization, concealment of treatment allocation, blinding, completeness of follow-up, and use of intention-to-treat analysis.

### Statistical Analysis

We assessed the overall effect of intensive control of glucose on the risk of incident stroke based on all the data from the nine trials. Individual trials relative risk (RRs) and 95% confidence intervals (CIs) were calculated from event numbers extracted from each trial before data pooling. Both fixed-effected and random-effects model were used to evaluate the pooled RR for intensive glucose control compared with standard therapy. Although both models yielded similar findings, results from the random-effects models are presented here, which assumed that the true underlying effect varies among included trials. Furthermore, many investigators consider the random-effects model to be a more natural choice than the fixed-effects model in medical decision-making contexts [6], [7]. The percentage of variability across trials attributable to heterogeneity beyond chance was estimated with the I^2^ statistic [8], [9]. We explored potential heterogeneity in estimates of treatment effect with univariate meta-regression (for baseline characteristic of patients, such as baseline BMI, baseline glycosylated hemoglobin, and duration of follow-up). After this, a subgroup analysis was carried out based on publication year, number of patients, percentage male, mean age, BMI, total cholesterol, glycosylated hemoglobin, current disease, duration of follow-up, and Jadad score. We also did a sensitivity analysis by removing each individual trial from the meta-analysis. Egger test [10] was used to check for potential publication bias. All reported P values were two-sided and P values of less than 0.05 were regarded as significant for all included studies. Statistical analyses were carried out using software STATA (version 10.0).

## Results

**Figure 1:**
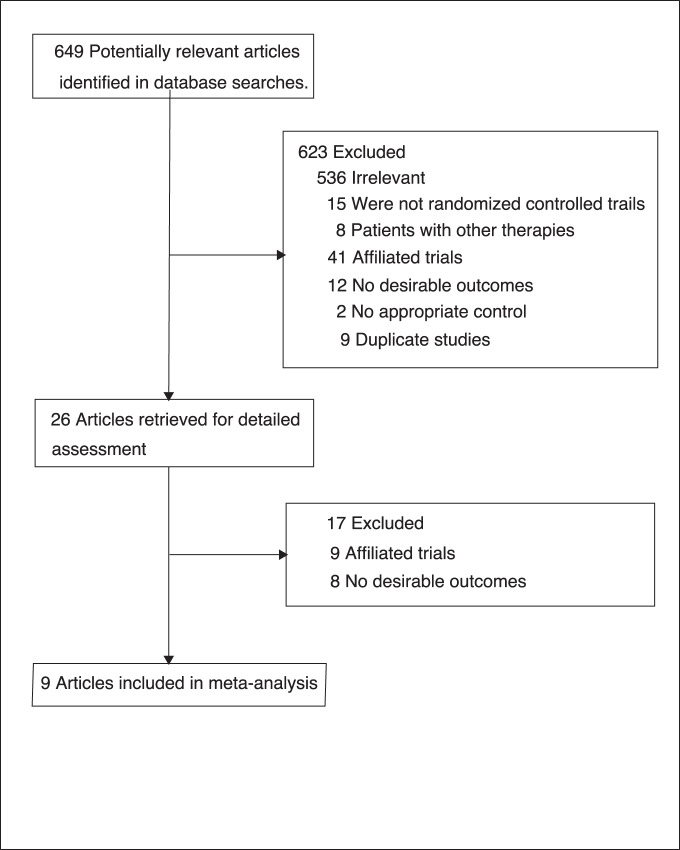
Flow diagram of the literature search and trials selection process.

We identified 649 articles from our initial electronic search, of which 623 were excluded during an initial review (title and abstract), we retrieved the full text for the remained 26 articles, and 9 [11]–[19] randomized controlled trials met the inclusion criteria (Figure 1 and Figure S1 [4]), which consisted of data of 59197 individual patients. Most other studies identified by our search did not provided relevant information, were not original investigators, or were duplicates of reports already identified. Table 1 summarized the characteristics of included studies and the important baseline information of the included patients. The trials included in this study compared intensive control of glucose with standard care. The trials had a sample size that ranged from 620 to 12537 patients, the mean age of the study patients ranged between 53.3 and 67.5 years, the mean BMI of the study patients ranged from 27.0 to 32.2 kg/m^2^, the mean total cholesterol of the study patients ranged between 178.2 and 210.0 mg/dL (four trials did not provided this information), the glycosylated hemoglobin of the study patients ranged from 5.8% to 9.4%, and the duration of follow-up ranged between 1 and 10.0 years. We restricted the inclusion criteria to randomized controlled trials with the number of events for stroke more than ten incident cases and a minimum of 12 months follow-up. Although the included trials scarcely reported on the key indicators of trial quality, the quality of the trials was also assessed according to the pre-fixed criteria using the Jadad score [5]. Overall, one trial [12] scored 5, five scored 4 [13], [14], [16], [17], [19], two scored 3 [15], [18], and the remaining one trial [11] scored 2.

**Table 1:** Design and characteristic of trials included in our meta-analysis.

Source	Publication year	No. of patients	Sex(male, %)	Mean (age, y)	BMI	Total cholesterol	Glycosylated hemoglobin	Current disease	Intervention	Follow-up(year)	Jadad score
DIGAMI Study Group [11]	1996	620	62.5	67.5	27.0	NG	8.1	DM and AMI	Insulin; conventional therapy	1	2
The NAVIGATOR StudyGroup [12]	2010	9306	49.4	63.8	30.5	210.0	5.8	impaired glucose tolerance/CVD/cardiovascular risk factors	Nateglinide; placebo	5	5
The VADT Investigators[13]	2009	1791	97.1	60.4	31.2	183.5	9.4	Type 2 DM	Intensive therapy;standard therapy	5.6	4
The ACCORD Study Group [14]	2008	10251	61.4	62.2	32.2	183.3	8.1	Type 2 DM	Intensive therapy;standard therapy	3.5	4
The ADVANCECollaborative Group [15]	2008	11140	57.5	66.0	28.0	NG	7.5	Type 2 DM	Intensive therapy; standard therapy	5	3
The PROactiveInvestigators [16]	2005	5238	66.5	61.7	30.9	NG	7.9	Type 2 DM and macrovasculardisease	Pioglitazone; placebo	2.9	4
The ORIGIN TrialInvestigators [17]	2012	12537	65.0	63.5	29.9	189.0	6.4	cardiovascular riskfactors plus impaired glucose/type 2 DM	Insulin glargine;standard care	6.2	4
The UKPDS Group [18]	1998	3867	61.0	53.3	27.5	178.2	7.1	Type 2 DM	Sulphonylureas or insulin; conventional therapy	10.0	3
The RECORD StudyTeam [19]	2009	4447	51.6	58.4	31.5	NG	7.9	Type 2 DM	Rosiglitazone; metformin and sulfonylurea	5.5	4

**Figure 2:**
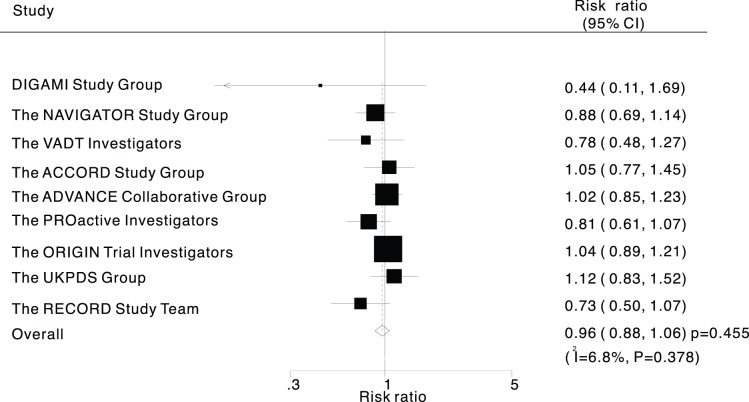
Effect of intensive control of glucose on risk of stroke.

Data for the effect of intensive control of glucose on the risk of incident stroke was available from all included trials, we noted that intensive control of glucose showed a 4% reduction in incident stroke, and with no evidence showed that intensive control of glucose protected against stroke risk (RR, 0.96; 95%CI: 0.88–1.06; P = 0.445, Figure 2). Although there was some evidence of heterogeneity across the trials included, a sensitivity analysis indicated that the results were not affected by sequential exclusion of any particular trial from all pooled analysis (data not shown).

**Figure 3:**
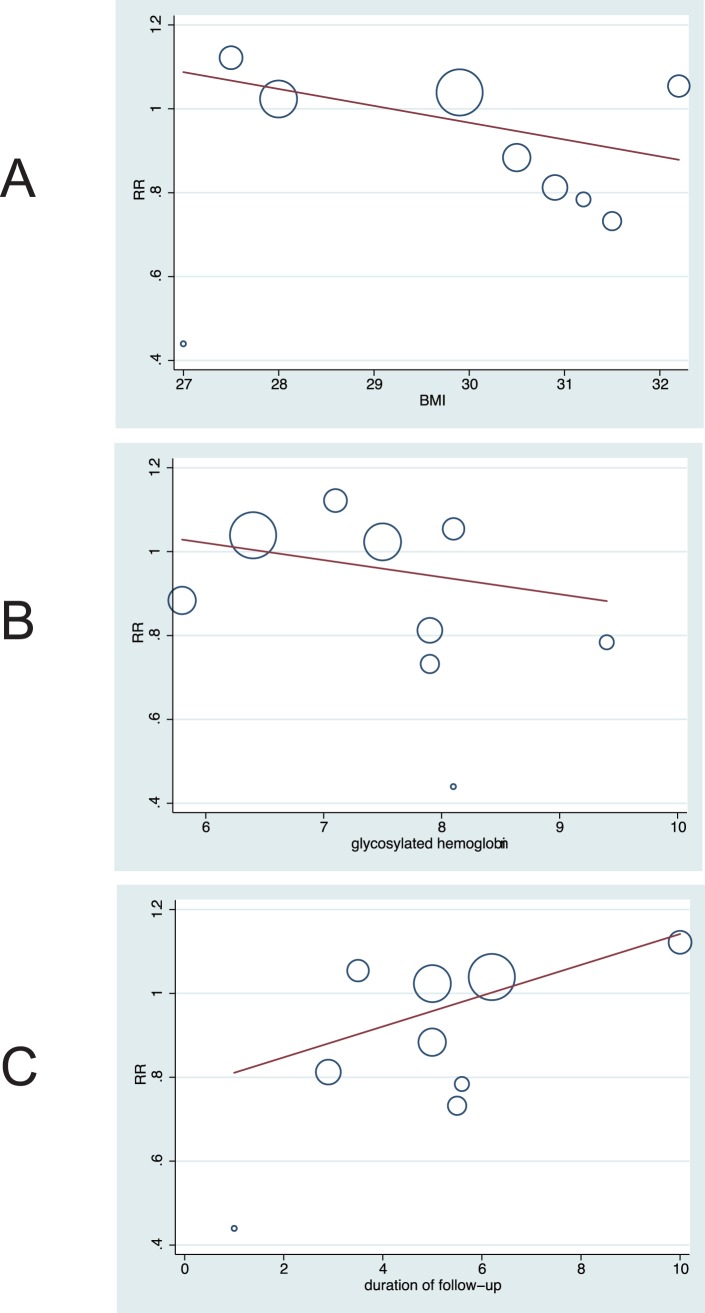
Meta-regression of (A: P = 0.242) baseline BMI, (B: P = 0.454) baseline glycosylated hemoglobin, and (C: P = 0.196) duration of follow-up for incident stroke.

Heterogeneity testing for analysis showed that P value are larger than 0.10, and we easy concluded that heterogeneity is not significant in the overall analysis, which suggesting that most variation was attributable to chance alone (Figure 2). In an exploratory attempt to identify other sources of the residual slight difference between trials, we undertook meta-regression analyses of baseline BMI, baseline glycosylated hemoglobin, and duration of follow-up (Figure 3). However, these variables did not seem to be important factors contributed to the association between intensive control of glucose and the risk of incident stroke.

Subgroup analyses were done for incident stroke, when we stratified the trials by baseline BMI, the RR for trials in BMI more than 30 was 0.86 (95%CI: 0.75–0.99; P = 0.041, Table 2), that for trials in BMI less than 30 was 1.04 (95%CI: 0.93–1.16; P = 0.497, Table 2). However, no other significant differences were identified between the effect of intensive control of glucose and standard care, based on additional subset factors (Table 2). Additionally, we used Egger test [10] to check for potential publication bias, which showed no evidence of publication bias for the outcomes of stroke (P value for Egger test, 0.301).

**Table 2:** Subgroup analyses of stroke.

Group	Relative Risks (RRs)and 95%CI	P value	Heterogeneity(%)	P value for heterogeneity
**Publication year**				
After 2005	0.96 (0.87–1.05)	0.358	5.0	0.389
Before 2005	0.89 (0.40–1.97)	0.775	43.8	0.182
**Number of patients**				
≥5000	0.98 (0.90–1.08)	0.749	0	0.498
<5000	0.86 (0.65–1.15)	0.316	35.0	0.202
**Percentage male (%)**				
≥60	0.99 (0.87–1.11)	0.817	8.1	0.365
<60	0.92 (0.78–1.09)	0.333	26.3	0.257
**Mean age (year)**				
≥62	1.01 (0.91–1.11)	0.922	0	0.598
<62	0.87 (0.72–1.06)	0.171	23.9	0.268
**BMI (kg/m^2^)**				
≥30	0.86 (0.75–0.99)	0.041	0	0.626
<30	1.04 (0.93–1.16)	0.497	0	0.604
**Total cholesterol (mg/dL)**				
≥185	0.99 (0.86–1.14)	0.898	14.5	0.279
<185	1.03 (0.84–1.26)	0.769	0	0.461
**Glycosylated hemoglobin (%)**				
≥8.0	0.92 (0.68–1.24)	0.576	11.6	0.323
<8.0	0.96 (0.87–1.07)	0.499	19.9	0.283
**Current disease**				
Typle 2 DM	0.95 (0.83–1.08)	0.414	14.4	0.322
DM or cardiovascular risk factors	0.97 (0.82–1.15)	0.727	22.5	0.275
**Duration of follow-up (year)**				
≥5	0.98 (0.89–1.09)	0.735	6.0	0.378
<5	0.89 (0.69–1.15)	0.377	21.5	0.280
**Jadad score**				
4 or 5	0.93 (0.83–1.04)	0.217	12.8	0.333
<4	1.04 (0.89–1.21)	0.648	0	0.397

## Discussion

Recently, evidence from large-scale randomized controlled trials [12], [17] has shown that intensive control of glucose is not significantly more effective than standard care in reducing the rate of stroke. This large quantitative review, including more than 59197 individuals with a broad range of baseline characteristics, suggested that with intensive control of glucose were at slightly reduced risk of incident stroke compared with those assigned standard care, however, this differences was not associated with a clinically and statistically significant. Additionally, our meta-analysis provides coherent evidence that intensive control of glucose can significantly reduce the risk of incident stroke when patients BMI more than 30. Although previous trials and meta-analysis [3] reported that the overall effect and stratified effect of intensive control of glucose on incident stroke was not significant.

The relation between lower glucose level and the risk of stroke was described initially by observational studies, which may overestimate the effect of this relationship. Previous meta-analysis of epidemiologic studies [20] suggested that reduced glucose level could lower the risk of coronary heart disease, ischaemic stroke, haemorrhagic stroke, and unclassified stroke. However, although KK Ray et al [3] concluded that intensive control of glucose significantly reduced coronary heart disease, and non-fatal myocardial infarction, it also concluded that intensive control of glucose did not significantly contribute to incident stroke. We therefore carried out a comprehensive systematic review and meta-analysis based on randomized controlled trials to explain the possible effect of intensive control of glucose on incident stroke.

Results from previous meta-analysis [21]–[23] already demonstrated that lipids, blood pressure, homocysteine had a clear effect on the risk of incident stroke. The relation between glucose level and cardiovascular outcomes also already illustrated [3]. However, these results do not prove that intensive control of glucose could reduce the risk of incident stroke, although this possibility already be considered. In addition, the results of our meta-analysis suggest that intensive control of glucose does not effect on the incidence of stroke. The potential explanation for this absence of difference could be that the association between glucose level and incident stroke may be reduced or balanced by the residual confounding factors, such as lipid level, blood pressure, and homocysteine level.

Subgroup analysis revealed that the risk of stroke were significantly reduced by intensive control of glucose compared with standard care when individuals with BMI more than 30. One potential reason for this could be that obesity patients always with other therapy or altered dietary because of high lipid level, or blood pressure [24], these variables also contributed an important role on the risk of incident stroke. Another potential explanation is that the association between lower glucose and incident stroke is due to glucose concentration always enhance blood viscosity, which could increase the risk of vascular complications.

Glycosylated hemoglobin may play an important role in the risk of stroke [25]–[28], although our study concluded that intensive control of glucose does not effect on stroke based on different glycosylated hemoglobin level, the extent of glycosylated hemoglobin lowering was unclear owing to the lack of data, we were unable to explore the association between the levels of glycosylated hemoglobin and incident stroke.

Diabetes mellitus is a metabolic disease that is diagnosed on the basis of sustained hyperglycemia. People with diabetes mellitus are at elevanted risk for incident stroke [29]. Previous meta-analysis based on epidemiologic studies [30] indicated the incidence of stroke is directly associated with the degree of hyperglycemia. However, our study also supported that intensive control of glucose had no effect on incident stroke in patients with diabetes mellitus (RR, 0.95; 95%CI: 0.83–1.08; P = 0.414). The reason for this could be that observational studies may overestimate the size of the effect.

In this meta-analysis, benefits was mainly detected in the prevention of incident stroke when patients with BMI more than 30. However, no other significant differences were detected between intensive control of glucose and standard care. Previous meta-analysis [3] has illustrated that the risk of stroke is not significantly reduced using intensive control of glucose compared with standard care. This conclusion was similar to our current meta-analysis. In our study, subgroup analysis indicated that intensive control of glucose contributed a causal relationship with the risk of stroke when patients BMI more than 30. The results of this meta-analysis are promising because the outcomes favor the use of intensive control of glucose interventions in obesity patients (BMI>30).

The limitations of our study are as follows: (1). The extent of glycosylated hemoglobin lowering was unclear, which restricted us to explore any correlation between glycosylated hemoglobin and incident stroke. (2). The association between different type of stroke and intensive control of glucose was not evaluated, because individual trials could not providing these data. (3). Our study was that the result is based on published data, where individual patients data and original data were not available, which limit the capacity to fully explore effects in stratified analysis.

For future trials, the type of stroke should be recorded and reported normatively, and it should be evaluated in any future trial. Furthermore, the extent of glycosylated hemoglobin lowering also be reported normatively. Finally, the role of intervention duration and dosage should be should be taken into consideration before evaluating clinical outcomes.

## Supporting Information

Figure S1 PRISMA Flowchart.
(DOC)

Table S1 PRISMA Checklist.
(DOC)
