# Supplementary figures and images for: Efficacy of Intensive Control of Glucose in Stroke Prevention: A Meta-Analysis of Data from 59197 Participants in 9 Randomized Controlled Trials
Source: PLoS One. 2013 Jan 23;8(1):e54465. doi: 10.1371/journal.pone.0054465 (PMC3553082; doi:10.1371/journal.pone.0054465)

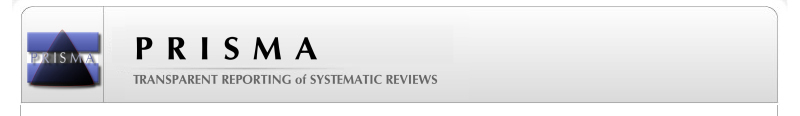
**PRISMA 2009 Flow Diagram**

**Screening**

**Included**

**Eligibility**

**Identification**

Supplement: Figure S1 — PRISMA Flowchart. (DOC) [file pone.0054465.s001.doc]
